# Morphology, Structure, and Ontogeny of Trichomes of the Grape Genus (*Vitis*, Vitaceae)

**DOI:** 10.3389/fpls.2016.00704

**Published:** 2016-05-25

**Authors:** Zhi-Yao Ma, Jun Wen, Stefanie M. Ickert-Bond, Long-Qing Chen, Xiu-Qun Liu

**Affiliations:** ^1^Key Laboratory of Horticultural Plant Biology, College of Horticulture and Forestry Science, Huazhong Agricultural University, Ministry of EducationWuhan, China; ^2^Department of Botany, National Museum of Natural History, MRC166, Smithsonian InstitutionWashington, DC, USA; ^3^UA Museum of the North Herbarium and Department of Biology and Wildlife, University of Alaska FairbanksFairbanks, AK, USA

**Keywords:** *Vitis*, trichome, morphology, structure, ontogeny, SEM, TEM

## Abstract

Trichomes are widely distributed on surfaces of different organs in the grape genus *Vitis* and are of taxonomic utility. To explore the morphology, structure and ontogeny of *Vitis* trichomes, we investigated the diversity and distribution of trichomes in 34 species of *Vitis*. Two main types of trichomes in *Vitis* are documented: non-glandular and glandular. Within non-glandular trichomes, ribbon and simple trichomes are found on different vegetative plant organs. The morphology and ontogeny of these types of trichomes are further examined with light microscopy and scanning electron microscopy. The ultrastructure of the glandular trichomes is explored with transmission electron microscopy. The ribbon trichomes are twisted, greatly elongated and unicellular, and this trichome type may be a morphological synapomorphy of *Vitis* and its closest tropical relative *Ampelocissus* and *Pterisanthes* in Vitaceae. The simple trichomes are documented in most species sampled in the genus. The glandular trichomes are multicellular, non-vascularized and composed of both epidermis and subjacent layers. We show that prickles occurring along the stems and petioles of *Vitis davidii* are modified glandular trichomes. We observed that glandular trichomes of *V. romanetii* secrete mucilage and volatile substances which trap insectes on the glands. Transmission electron microscopy indicates that metabolic products accumulate in vacuoles, the cytoplasm and intercellular spaces. We infer that glandular trichomes and young prickles are involved in the secretion of these metabolic products and the intercellular spaces may be the places of temporary storage of these secretions.

## Introduction

Trichomes usually originate from epidermal cells and are ubiquitous in many plant families, showing great diversity in morphology, cellular structure and function (Uphof, 1962). These epidermal appendages are unicellular or multicellular, branched or unbranched, glandular or non-glandular (Levin, 1973; Werker, 2000; Yang and Ye, 2013). They have been observed on all vegetative and reproductive organs in angiosperms (Werker, 2000; Wagner et al., 2004). These special structures can protect from herbivory by large mammals, insect and pathogens attacks, increase light reflectance, regulate temperature and decrease water loss (Levin, 1973; Wagner et al., 2004).

Grapes have been widely recognized for their agronomic and economic importance as fresh fruits, and sources of wine and raisins (Wen, 2007). Other species of the grape genus (*Vitis* L.) are important germplasm resources for the wine grape (*V. vinifera* L.), and have been bred to improve resistance of cultivated varieties against many fungal diseases and to enhance cold tolerance (Staudt and Kassemeyer, 1995; Wang et al., 1995; Staudt, 1997; Brown et al., 1999; Wan et al., 2007; Zhang et al., 2012; Gerrath et al., 2015).

*Vitis* includes ca. 70 species mostly in the temperate regions of the Northern Hemisphere (Chen et al., 2007; Wen, 2007; Zecca et al., 2012; Moore and Wen, 2016). *Vitis* has been shown to be part of the *Ampelocissus*–*Vitis* clade, one of the five major clades in Vitaceae (Soejima and Wen, 2006; Liu et al., 2013, 2016; Wen et al., 2013). The genus *Vitis* has been widely studied over the past 10 years and confirmed to include two subgenera (subgenus *Muscadinia* and subgenus *Vitis*) (Mullins et al., 1992; Soejima and Wen, 2006; Aradhya et al., 2008; Tröndle et al., 2010; Péros et al., 2011; Zecca et al., 2012; Miller et al., 2013; Wan et al., 2013; Liu et al., 2016). Subgenus *Muscadinia* includes only two species: *V. popenoei* J. H. Fennel from Mexico and *V. rotundifolia* Michx. from the south-eastern U.S.A. (Brizicky, 1965; Moore, 1991; Wen, 2007), while, subgenus *Vitis* includes the Eurasian and the New World clades (Péros et al., 2011; Zecca et al., 2012; Wan et al., 2013; Liu et al., 2016). The phylogeny and taxonomy within the subgenus *Vitis* have not been well resolved due to the highly similar morphology, overlapping distribution, introgression and interspecific hybridization among species within the clade (Aradhya et al., 2008; Moore and Wen, 2016). Many *Vitis* species are differentiated and delimited mainly by the types, distribution and density of trichomes (Moore, 1991; Chen et al., 2007).

However, trichomes of *Vitis* have rarely been studied systematically. Detailed documents of trichome types and distribution in vegetative parts of *Vitis* have not been shown in previous studies (Galet, 1988; Moore, 1991; Chen et al., 2007). He et al. (1994) and Cheng et al. (2013) have explored trichome diversity and some anatomy in *Vitis* trichomes. Nonetheless, they did not show clear and detailed anatomical structures and distribution of trichomes and did not perform any developmental studies of trichomes. The lack of structural and ontogenetic studies on *Vitis* trichomes has limited the exploration of their function and the significance as the basis for inferring the evolutionary relationships among the species in *Vitis*.

*Vitis davidii* is the only species with prickles on stems and petioles in *Vitis*. The unique character was considered to distinguish *V. davidii* from other species in *Vitis* (Galet, 1988; Chen et al., 2007). However, very little is known about its structure, ontogeny, function and the relationship with other trichome types.

The primary purpose of this study is to: (1) document types and distribution of *Vitis* trichomes to provide available and reliable information for taxonomy, and (2) to explore the anatomical structure, ultrastructure and ontogeny of *Vitis* trichomes, especially prickles of *V. davidii*, to infer the relationship among trichomes and prickles.

## Materials and methods

### Plant materials

This study sampled 34 *Vitis* species covering three different distribution areas: 27 from Asia, six from North America and one from Europe (Table 1).

**Table 1:** **The distribution of trichomes on different vegetative organs in *Vitis***.

**Species**		**Plant parts**									**Number of plants**
		**Current year's branchlets**	**Young leaves**	**Mature leaves**							
				**Petioles**	**Leaf surfaces**					**Leaf margins**	
					**Adaxial**		**Abaxial**				
					**Leaf epidermis**	**Leaf veins**	**Leaf epidermis**	**Leaf veins**	**Leaf vein axils**		
**ASIAN SPECIES**											
1	*V. adenoclada*^123^	*G+* *R*	*G* *R+* *S*	*G* *R*		*R* *S*	*R+*	*R* *S*			3
2	*V. amurensis*	*R*	*R+* *S*	S^*^				R^*^ S^*^	*S*		4
3	*V. balansana*	*R*	*R+*				R^*^				3
4	*V. bellula* var. *pubigera*	*R*	*R+* *S*	*S*		*S*	*R+*	*R* *S*		*S*	3
5	*V. betulifolia*	*R*	*R+* *S*	*R*		*R* *S*	*R*	*R* *S*			1
6	*V. bryoniifolia*	*R*	*R+* *S*	*R* *S*		*R* *S*	*R*	*R* *S*		*S*	4
7	*V. chunganensis*^123^	G^*^ R^*^	G^*^ R^*^	G^*^				G^*^			3
8	*V. chungii*		R^*^ S^*^			S^*^		S^*^		S^*^	5
9	*V. davidii*^123^	*P+*	*P* *R* *S*	*P*		*S*		*R* *S*	*S*	*S*	3
10	*V. erythrophylla*		*R* *S*	*S*		*R* *S*		*R* *S*		*S*	3
11	*V. flexuosa*^1^	*S*	*R* *S*	*S*		*S*		*S*	*S*		5
12	*V. hancockii*^1^	*S+*	*R* *S+*	*R* *S+*		*S*		*R* *S+*	*S*	*S*	4
13	*V. heyneana*	*R*	*R+* *S*	*R*		*R* *S*	*R+*	*R* *S*			3
14	*V. heyneana* subsp. *ficifolia*	*R*	*R+* *S*	*R*		*R* *S*	*R+*	*R* *S*			3
15	*V. hui*	*R* *S*	*R+* *S*	*R* *S*	*S+*	*R* *S*	*R+*	*R* *S*		*S*	3
16	*V. lanceolatifoliosa*	*R*	*R+* *S*	*R*		*R* *S*	*R+*	*R* *S*			3
17	*V. piasezkii*	R^*^ S^*^	*R* *S*	R^*^ S^*^	*S+*	*S*	R^*^	*S+*		*S*	3
18	*V. piasezkii* var. *pagnucii*		*R* *S*	*S*		*S*		*R* *S*	*S*		3
19	*V. pseudoreticulata*	*R*	*R+* *S*	*R* *S*		*R* *S*		*R* *S*	*S*	S^*^	5
20	*V. retordii*^12^	*R*	*R+* *S+*	*R* S^*^	*S+*	*S*	*R+*	*R* *S*		*S*	3
21	*V. romanetii*^123^	*G+* *R* *S+*	*G+* *R+* *S+*	*G* *R* *S+*		*S*	*R*	*G* *R* *S+*		*S*	3
22	*V. ruyuanensis*		*S*	*S*		*S*		*S*		*S*	4
23	*V. shenxiensis*^12^	*G* *R*	*G* *R* *S*	*G* *R* *S*		*S*		*S*	*S*	*S*	1
24	*V. sinocinerea*	*R* *S*	*R+* *S*	*R* *S*		*S*	*R+*	*R* *S*		*S*	3
25	*V. tsoii*	*S*	*R* *S*	*R* *S*		*R* *S*	R^*^	*R* *S*		*S*	3
26	*V. wilsoniae*^12^	*R*	*R+* *S*	*R* *S*		*S*		*R* *S*	*S*	*S*	3
27	*V. wuhanensis*	*R*	*R+* *S*	*R* *S*		*R* *S*		*R* *S*		*S*	3
**EUROPEAN SPECIE**											
28	*V. vinifera*	*R*	*R* S^*^	*R* S^*^			R^*^	*R* S^*^			4
**NORTH AMERICAN SPECIES**											
29	*V. labrusca*	G^*^ R	*R+* *S*	G^*^ R		*S*	*R+*	*R* *S*		*S*	1
30	*V. mustangensis*	*R*	*R+*	*R*			*R+*	*R*			1
31	*V. riparia*		*S*	*S*		*S*		*S*	*S*	*S*	1
32	*V. rupestris*		*R* *S*	*S*		*S*		*S*	*S*	*S*	1
33	*V. shuttleworthii*	*R*	*R+*	*R*			*R+*	*R*			1
34	*V. rotundifolia*	G^*^ S	*R* *S*	*S*		*S*		*S*	*S*		1

*Abbreviations in table: R, ribbon trichomes; G, glandular trichomes; P, prickles; S, simple trichomes. The blank spot indicate that the organ is glabrous. The numbers “1,” “2,” “3” indicate the species were examined with light, scanning electron and transmission electron microscopy respectively. “+” indicate that the trichome type is stable present and forms remarkable dense indumentum on the vegetative organ*.

“*” *indicate that the trichome type is variable and not stable present on the vegetative organ. The unmarked indicate that the trichome type is stable present, but not forms remarkable dense indumentum on the vegetative organ*.

### Stereomicroscope

Herbarium specimens and fresh vegetative parts (current year's branchlets, young leaves, petioles, adaxial and abaxial leaf surfaces of mature leaves, and leaf margins) of *Vitis* were examined to characterize the types and distribution of trichomes in 34 *Vitis* species using a Nikon SMZ800 stereomicroscope. At least three plants were sampled for most species, except *V. betulifolia*, *V. shenxiensis* and all North American species for which there was only one individual sampled. Young leaves were taken from shoot tips, while mature ones were taken from nodes after the 9th node (counting from the top) to assure their mature condition. Five young and five mature leaves from each plant were sampled respectively. The presence/absence and types of trichomes on different vegetative organs were documented.

The detailed anatomy, ontogeny and ultrastructure studies of *Vitis* trichomes were conducted using light microscopy (LM), scanning electron microscopy (SEM) and transmission electron microscopy (TEM).

### Light microscopy

To examine the anatomical structure of trichomes, 33 samples of stems, petioles and leaf mid-veins, from nine *Vitis* species (*V. adenoclada*, *V. chunganensis*, *V. davidii*, *V. flexuosa*, *V. hancockii*, *V. retordii*, *V. romanetii*, *V. shenxiensis*, and *V. wilsoniae*), were fixed in 70% FAA for 24 h. Samples were dehydrated in an ethanol series (Johansen, 1940) and embedded in paraffin wax. Transversal and longitudinal sections of 6–8 μm were cut with a Leica RM2235 rotary microtome, mounted on slides and stained with 1% safranin O and 0.5% fast green.

### Scanning electron microscopy

Thirty-eight samples including stems, petioles and leaves from representatives of eight species (*V. adenoclada*, *V. chunganensis*, *V. davidii*, *V. heyneana*, *V. retordii*, *V. romanetii*, *V. shenxiensis*, and *V. wilsoniae*) were fixed in 2.5% glutaraldehyde in 0.1M phosphate buffer, pH7.2. The fixed samples were dehydrated in an ethanol series and critical point dried. Samples were placed with double-sided tape on stubs and sputter-coated with a thin gold layer (Robards, 1978). The types and morphology of trichomes were examined with a JEOL JSM-6390LV Scanning Electron Microscope. Based on the SEM images, we measured the heights/lengths of different trichome types and the sizes of parts of glandular trichomes or prickles.

### Transmission electron microscopy

Four samples of glandular trichome or young prickle from *V. adenoclada*, *V. chunganensis*, *V. davidii*, and *V. romanetii* were fixed in 1% osmium tetroxide, in 0.1M phosphate buffer, pH7.2, and processed following standard techniques (Roland and Vian, 1991). Ultra-thin sections of both glandular trichomes and the prickles were made and viewed under a Hitachi H-7650 Transmission Electron Microscope.

### Histochemical lignin estimation

The histochemical lignin tests for prickles of different growth stages of *V. davidii* were performed in fresh hand-cut sections. The sections were soaked in a fresh phloroglucinol solution, and then a few drops of 35% HCl were added (Pillonel et al., 1991). The positive result will shows an intense pink color.

## Results

### Trichome types

We used the terminology of Payne (1978) to describe the trichomes of *Vitis* in this study. Two general trichome types in *Vitis* are documented: non-glandular and glandular. Non-glandular trichomes include ribbon and simple trichomes.

### Non-glandular trichomes

**Figure 1:**
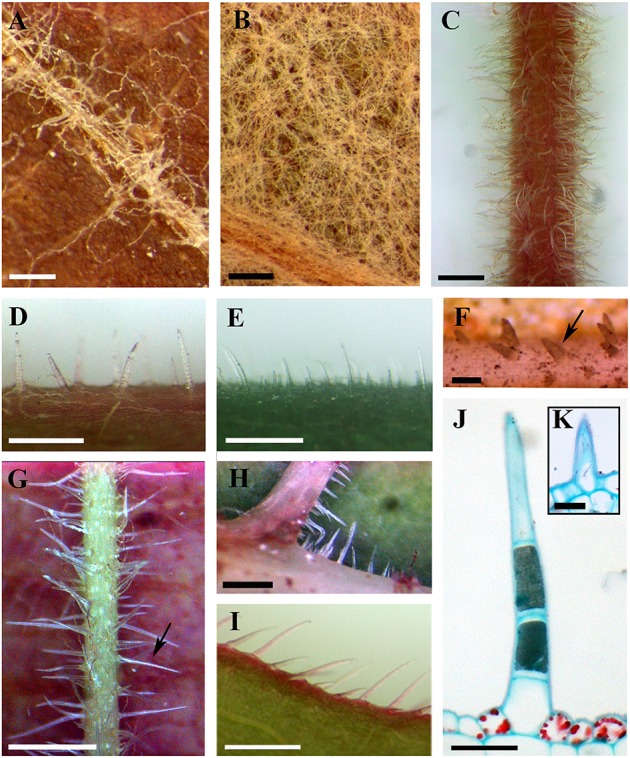
**Distributions and structure of ribbon and simple trichomes**. **(A,B)** Ribbon trichomes on different plant organs. Ribbon trichomes on adaxial leaf surfaces of *V. bryoniifolia* **(A)** and on abaxial leaf surfaces of *V. adenoclada* **(B)**. **(C–I)** Simple trichomes on different plant organs. Simple trichomes on branchlets of *V. hancockii* **(C)**, petioles of *V. pseudoreticulata* **(D)**, adaxial leaf surfaces of *V. retordii* **(E)**, adaxial leaf veins **(F)** and abaxial leaf veins of *V. hancockii* **(G)**, abaxial leaf vein axils of *V. shenxiensis* **(H)** and leaf margins of *V. piasezkii* **(I)**. Arrow in **(F)** shows a simple unicellular trichome and in **(G)** shows a simple uniseriate multicellular trichome. **(J)** Photomicrograph of microtome section of a simple trichome with uniseriate cells of *V. hancockii*. **(K)** A simple unicellular trichome of *V. davidii*. Scale bars: **(A,B)** = 0.25 mm; **(C,E)** = 1 mm; **(D,G–I)** = 0.5 mm; **(F)** = 100 μm; **(J)** = 50 μm; **(K)** = 20 μm.

In *Vitis* the distribution and densities of non-glandular trichomes vary among different vegetative organs including current year's branchlets, young leaves and mature leaves, as well as on petioles, adaxial and abaxial leaf surfaces and leaf margins (Figures 1A–I). Almost all species sampled bear the ribbon and simple trichomes on different plant organs (Table 1).

**Figure 2:**
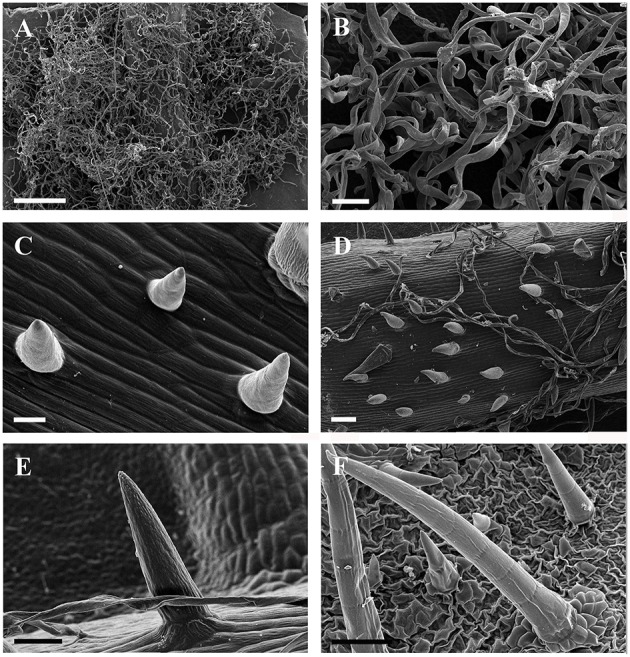
**SEM micrographs of ribbon and simple trichomes. (A,B)** Ribbon trichomes on abaxial leaf surfaces of a young leaf in *V. wilsoniae*. **(C–F)** Simple trihcomes: **(C)** young simple trichomes on veins of *V. romanetii*; **(D,E)** simple trichomes on veins of young leaves in *V. davidii*; **(F)** simple trichomes on adaxial leaf surfaces of *V. retordii*. Scale bars: **(A)** = 500 μm; **(B,E)** = 50 μm; **(C)** = 20 μm; **(D,F)** =100 μm.

Ribbon trichomes are flattened, slender, greatly elongated, twisted and unicellular (Figures 1A,B, 2A,B). Ribbon trichomes can be as long as several millimeters. In total, 32 of the 34 species sampled bear the ribbon trichomes (Table 1). Twenty-three of the 34 species sampled have ribbon trichomes on current year's branchlets. On mature leaves, we observed ribbon trichomes on the petioles of 21 of the 34 species sampled. In addition, on the adaxial leaf surfaces of some species (e.g., *V. adenoclada* and *V. bryoniifolia*) only the leaf veins bear sparse ribbon trichomes (Figure 1A; Table 1). On the abaxial leaf surfaces, ribbon trichomes are found in 26 species, of which 11 species have non-deciduous dense arachnoid indumentum composed of this trichome type (Figure 1B; Table 1; *V. adenoclada*, *V. bellula* var. *pubigera*, *V. heyneana*, *V. heyneana* subsp. *ficifolia*, *V. hui*, *V. lanceolatifoliosa*, *V. retordii*, *V. sinocinerea*, *V. labrusca*, *V. mustangensis*, and *V. shutterworthii*).

Simple trichomes are unbranched, uniseriate, or unicellular (Figures 1C–J, 2C–F), and originate from the epidermis (Figure 2C). Simple trichomes are found in 30 of the 34 *Vitis* species examined, but their distribution on different plant organs varies (Table 1). On mature leaves, only eight species have simple trichomes on the current year's branchlets (Figure 1C), and 22 species have this trichome type on petioles (Figure 1D), while 28 species bear simple trichomes on adaxial leaf surfaces (Figures 1E,F). Simple trichomes are present on the veins of abaxial leaf surfaces in 30 species (Figure 1G), and 11 species (*V. amurensis*, *V. davidii*, *V. flexuosa*, *V. hancockii*, *V. piasezkii* var. pagnucii, *V. pseudoreticulata*, *V. shenxiensis*, *V. wilsoniae*, *V. riparia*, *V. rupestris*, and *V. rotundifolia*) bear simple trichomes on the abaxial leaf vein axils (Figure 1H). On leaf margins, 20 species bear simple trichomes (Figure 1I). We observed dramatic length differences in simple trichomes ranging from 50–200 μm in length on veins of adaxial and/or abaxial leaf surfaces in *V. adenoclada*, *V. davidii*, *V. hancockii*, *V. heyneana*, and *V. wilsoniae* (Figures 1F, 2D,E), while some of simple trichomes are up to ca. 750–900 μm long in *V. romanetii* and *V. retordii* (Figure 2F).

**Figure 3:**
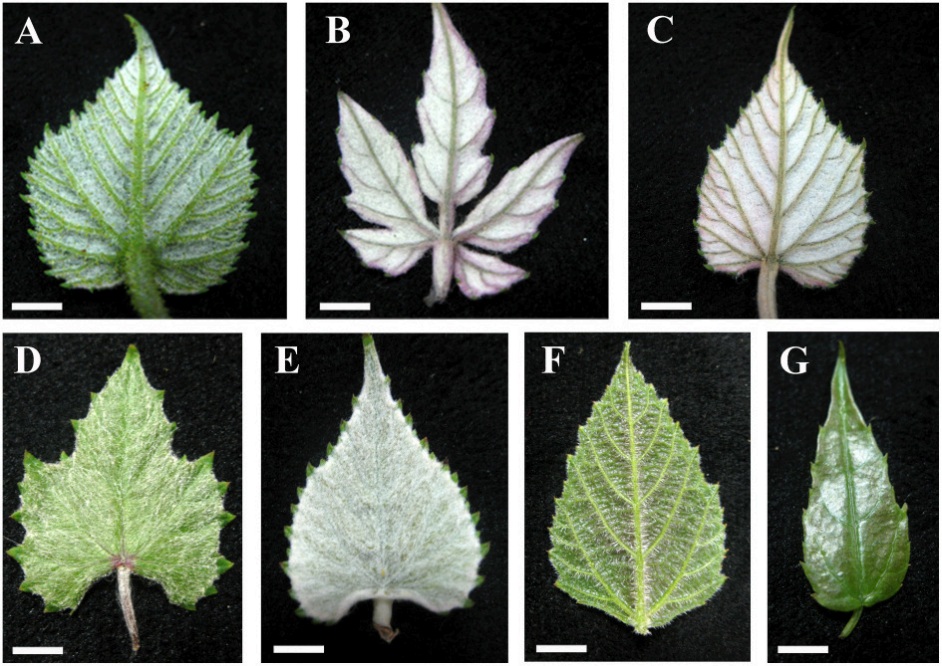
**Young leaves with ribbon and/or simple trichomes**. **(A–E)** Dense ribbon trichomes on abaxial leaf surfaces of *V. romanetii* **(A)**, *V. lanceolatifoliosa* **(B)** and *V. retordii* **(C)** and adaxial leaf surfaces of *V. wuhanensis* **(D)** and *V. wilsoniae* **(E)**. **(F)** Dense simple trichomes on abaxial leaf surfaces of *V. hancockii*. **(G)** *V. chungii* nearly glabrous on the abaxial leaf surfaces. Scale bars: **(A–E,G)** = 3 mm; **(F)** = 4 mm.

Almost all species examined (30/34) show an indumentum composed of ribbon trichomes on young leaves (Figures 3A–E), which may become sparse or glabrescent on mature leaves (Table 1). Ribbon trichomes are only occasionally observed on the young leaves of some species (*V. chunganensis* and *V. chungii*) (Table 1). Thirty species sampled were covered with simple trichomes on young leaves (Figure 3F; Table 1) and some species sampled are nearly glabrous (Figure 3G). Twenty-eight species sampled bear both ribbon and simple trichome types (Figure 3A, Table 1).

The non-glandular trichome density, especially the ribbon trichome density, changed on leaves of some species of *Vitis* (e.g., *V. amurensis* and *V. wilsoniae*) during development of leaves. The ribbon trichomes become lost with development of the leaves in some species of *Vitis*.

### Glandular trichomes and prickles

**Figure 4:**
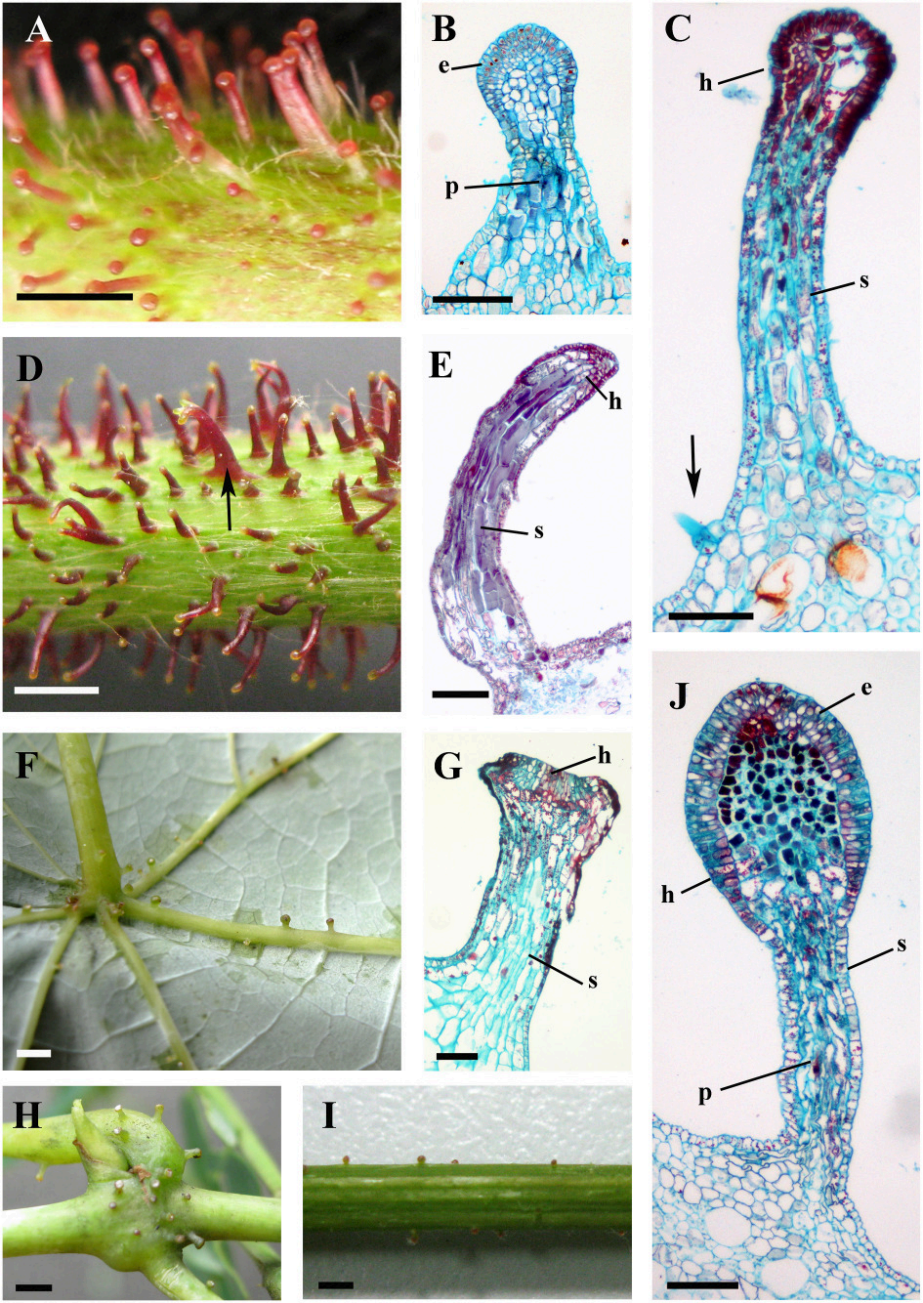
**Structures and distributions of glandular trichomes in ***Vitis*****. **(A–C)** Glandular trichomes of *V. romanetii*: **(A)** glandular trichomes on stems; **(B,C)** structures of glandular trichomes and simple trichomes on the epidermis of stalks of glandular trichomes (arrow). **(D,E)** Glandular trichomes on the stems of *V. adenoclada*: glandular trichomes on stems and some of these trichomes form complexes (arrow) **(D)**. **(F–H)** Glandular trichomes of *V. chunganensis*: glandular trichomes on basal parts of veins of abaxial leaf surfaces **(F)** and nodes **(H)**. **(I,J)** Glandular trichomes on stems of *V. shenxiensis*. Abbreviations: e, epidermal cells; p, parenchymatic cells; h, head; s, stalk. Scale bars: **(A,D,F,H,I)** = 1 mm; **(B,C,E,G,J)** = 100 μm.

**Figure 5:**
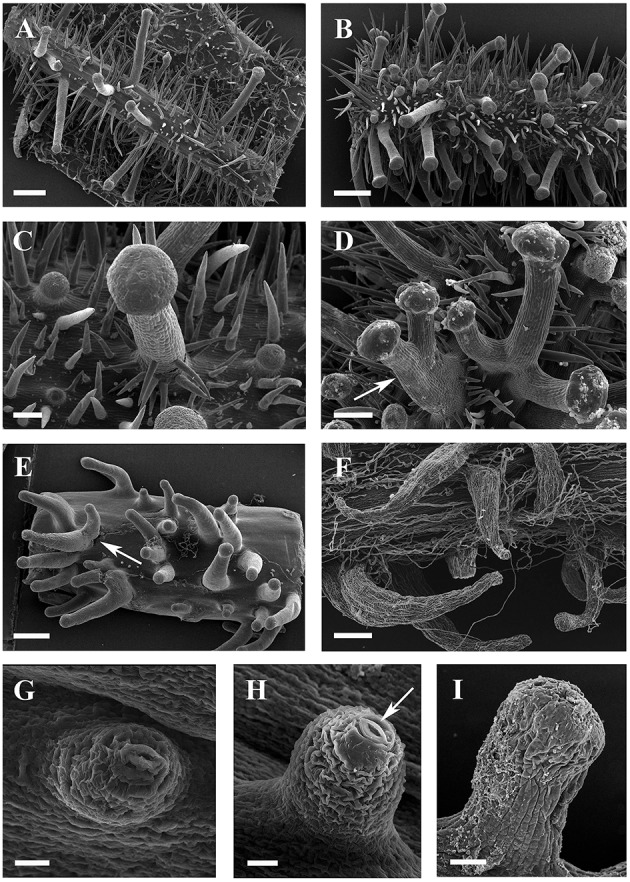
**SEM micrographs of glandular trichomes of ***Vitis***. (A,B)** Glandular trichomes and simple trichomes on abaxial leaf surfaces **(A)** and petioles **(B)** of *V. romanetii*. **(C)** Simple trichomes on the epidermis of stalks of glandular trichomes of *V. romanetii*. **(D)** Trichome complexes of glandular trichomes (arrow) of *V. romanetii*. **(E)** Complexes (arrow) of prickles on petioles of *V. davidii*. **(F)** Glandular trichomes with hooked stalks on stems of *V. adenoclada*. **(G,H)** Emerging cell masses **(G)** and a glandular head without stalk and an obvious stoma (arrow) on the top of glandular head **(H)** in *V. shenxiensis*. **(I)** A glandular trichome on nodes of *V. chunganensis*. Scale bars: **(A,B,E)** = 500 μm; **(C)** = 100 μm; **(D,F)** = 200 μm; **(G,H)** =20 μm; **(I)** = 50 μm.

Glandular trichomes are observed in six species (*V. romanetii*, *V. adenoclada*, *V. chunganensis*, *V. labrusca*, *V. rotundifolia*, and *V. shenxiensis*), but prickles are only observed in *V. davidii*. Glandular trichomes vary in shape, size, density, distribution and color among *Vitis* species in this study (Figures 4A–J, 5A–I). All of them are multicellular, non-vascularized and composed of both epidermis and subjacent layers (Figures 4B,E,G,J). The glandular trichomes of these species are composed of apparent glandular heads with multiple layers of epidermal cell and stalks with one layer of epidermal cell (Figure 4J). Stomata are frequently observed on the upper region of glandular heads in most *Vitis* species sampled except in *V. chunganensis* (Figures 5C,F,H,I).

**Figure 6:**
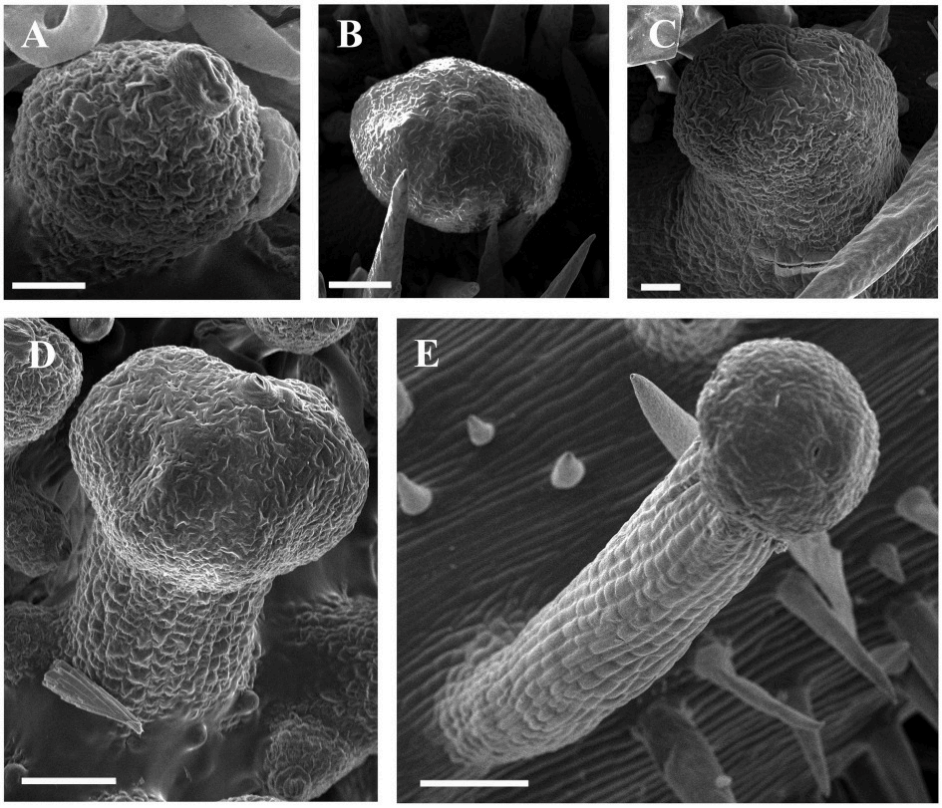
**SEM micrographs of development of glandular trichomes of ***V. romanetii***. (A–C)** Glandular heads without apparent stalks. **(D)** Young glandular trichome with short stalk. **(E)** Mature glandular trichome with long stalk. Scale bars: **(A)** = 20 μm; **(B)** = 50 μm; **(C)** = 20 μm; **(D)** = 50 μm; **(E)** = 100 μm.

**Figure 7:**
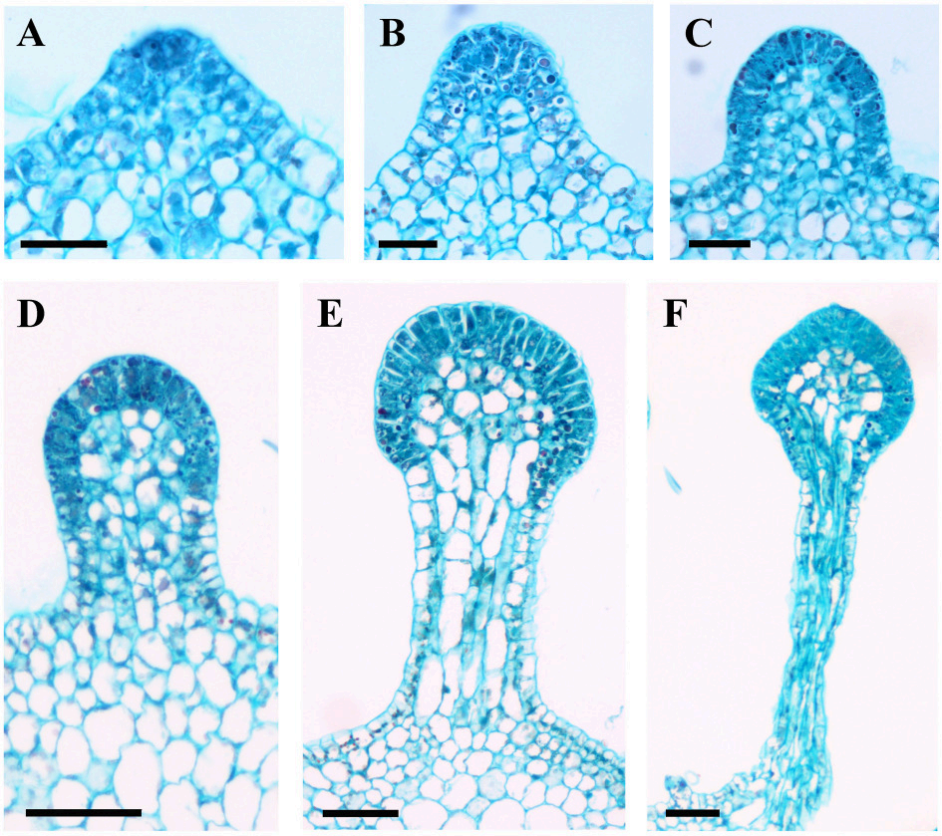
**Development of glandular trichomes of ***V. romanetii***. (A)** Emerging cell masses. **(B)** Original gland head with rapid proliferation and elongation of upper region epidermal cells. **(C)** Emerging glandular head without stalk. **(D)** Young glandular trichome with short stalk. **(E)** Elongation of stalk of young glandular trichome. **(F)** Mature glandular trichome. Scale bars: **(A–C)** = 50 μm; **(D–F)** = 100 μm.

Developmentally, we can recognize three stages of glandular trichome growth (Figures 6A–E, 7A–F). Initially, cell masses emerge from the epidermis where stomata may develop with rapid proliferation and elongation of upper region epidermal cells (Figures 5G, 7A,B), forming a sessile glandular head (Figures 5H, 6A–C, 7C). Secondly, rapid proliferation and expansion of epidermal cells and underlying parenchymatous cells cause a stalk to develop (Figures 6D, 7D,E). Finally, glandular trichomes fully mature (Figures 6E, 7F) and proceed to the senescence period in which they appear shrunken (Figure 5I).

The predominant distributions of dense glandular trichomes of *V. romanetii* are on branchlets, petioles and veins of abaxial leaf surfaces alongside dense and long simple trichomes (Figures 4A–C, 5A–D). The glandular trichomes are pink/yellow, erect and ca. 1200 μm long. Diameters of their glandular heads are ca. 200 μm. They emerge and continue to grow with the extension of stalks until maturity. Interestingly, adjacent glandular trichomes may produce trichome complexes which may be formed after fusion or branching, meanwhile, this similar structure is also observed in *V. adenoclada* and *V. davidii* (Figures 4D, 5D,**E**). Simultaneously, many simple trichomes are observed on the epidermis of the stalks of glandular trichomes (Figures 4C, 5C,D).

**Figure 8:**
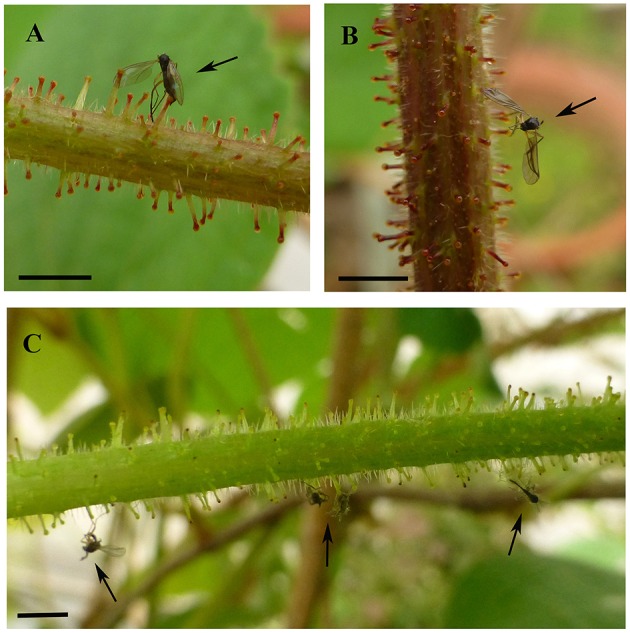
**Insects were glued to the glandular trichomes of ***V. romanetii*** (A–C)**. Arrows indicate stuck insects. Scale bars: **(A–C)** = 3 mm.

The glandular trichomes of *V. romanetii* are mucilaginous and emit volatile substance based on our observation. Many insects are often glued to their glands (Figures 8A–C).

Glandular trichomes of *V. adenoclada* have wine-red and hooked stalks and faint-yellow glandular heads (Figures 4D,E, 5F). They are commonly observed on branchlets and petioles. The height of these mature trichomes is up to 1100 μm and the diameter of the glandular heads is ca. 80–100 μm.

The glandular trichomes of *V. chunganensis* are present on basal veins of abaxial leaf surfaces, petioles and at along stem nodes (Figures 4F,H) and have short stalks and inverted trapezoid heads (Figures 4G, 5I). The height of these trichomes is only 80–550 μm and the diameter of the glandular heads is 100–350 μm.

Glandular trichomes of *V. shenxiensis* bear similar structures to those of *V. romanetii*, but are less dense on branchlets (Figures 4I,J). Furthermore, these trichomes are ca. 650 μm long and have large glandular heads with diameters up to 250 μm.

**Figure 9:**
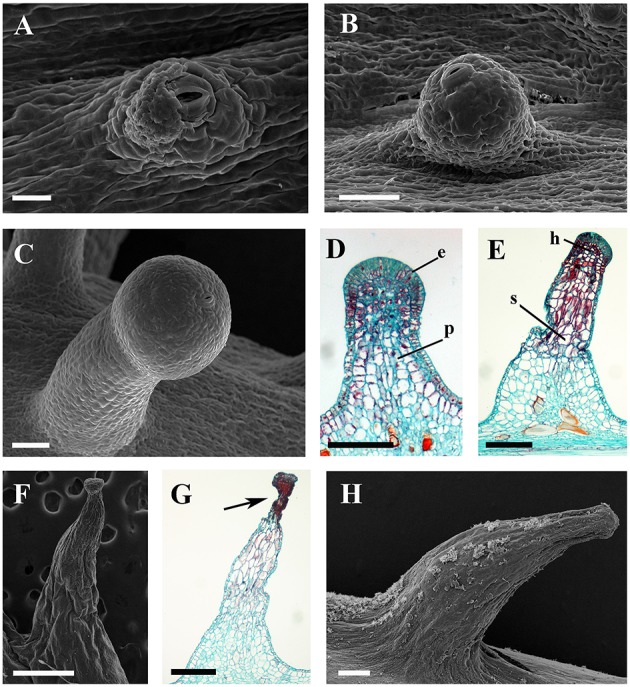
**Development and structure of prickles of ***V. davidii***. (A)** Emerging cell masses with a notable stoma and **(B)** a glandular head without stalk. **(C)** Young prickle with stalk. **(D)** Structure of the young prickle. **(E)** Mature prickle. **(F)** Shrinking prickle. **(G)** Senescent prickle, arrow indicating the shrinking top of gland. **(H)** The lignifying prickle. Abbreviations: e, epidermal cells; p, parenchymatic cells; h, head; s, stalk. Scale bars: **(A)** = 20 μm; **(B,C)** = 50 μm; **(D)** = 100 μm; **(E,H)** = 200 μm; **(F)** = 500 μm; **(G)** =250 μm.

**Figure 10:**
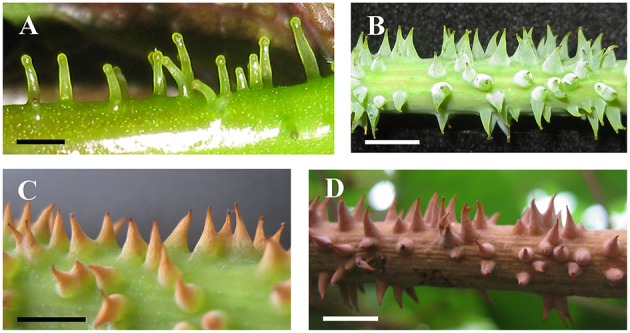
**Development and morphology of prickles of ***V. davidii***. (A)** Young prickles on petiole. **(B)** Mature prickles on stem. **(C)** The withering and lignifying terminal part of prickles. **(D)** Fully lignifying prickles. Scale bars: **(A)** = 1 mm; **(B–D)** = 5 mm.

The prickles of *V. davidii* are similar to those of other glandular trichomes in *Vitis*. They are also multicellular, non-vascularized and composed of both epidermis and subjacent layers. The prickles have stalks with one layer of epidermal cells and apparent glandular heads with multiple layers of epidermal cells on which stomata are frequently observed. Similar to early developmental stages of glandular trichomes, initially, the cell masses emerge from the epidermis with notable stomata at ca. 200 μm in diameter (Figure 9A); then the glandular heads grow without stalks (Figure 9B); and finally, the stalks of prickles form with rapid proliferation and expansion of epidermal and parenchymatous cells (Figures 9C,D, 10A). However, the prickles increase in both height and basal width during development (Figure 9E). They become conical and glaucous (Figure 10B) and lignified at maturity (Figure 10C and Supplementary Image 1). Some glandular heads of prickles shrink with lignification (Figures 9F,G). Finally, they become hard and prickly (positive result for histochemical lignin estimation) (Figures 9H, 10D and Supplementary Image 1).

### Ultrastructure

**Figure 11:**
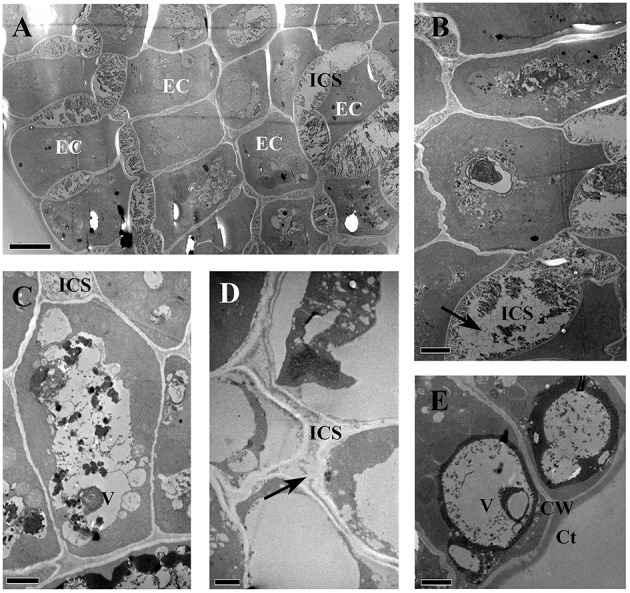
**TEM images of the glandular trichomes and young prickles. (A)** Epidermal cells of gland head in *V. davidii*, showing containing of dense thready and granular material in vacuoles and intercellular spaces. **(B)** Detail of the epidermal cells of *V. davidii*. Arrow indicate dense granular material. **(C)** Numerous irregular masses of osmiophilic material accumulating in vacuoles of epidermal cells of gland head in *V. adenoclada*. **(D)** Dense thready material (arrow) in the intercellular spaces of *V. chunganensis*. **(E)** Vacuoles are filled with osmiophilic material. Abbreviations: CW, cell wall; Ct, cuticle; EC, epidermal cells; ICS, intercellular spaces; V, vacuole. Scale bars: **(A)** = 5 μm; **(B,C,E)** = 2 μm; **(D)** = 1 μm.

The glandular heads of *V. davidii*, *V. adenoclada*, *V. chunganensis*, and *V. romanetii* showed similar ultrastructure. There are numerous osmiophilic granular and irregular masses found in the dense cytoplasm and vacuoles in the epidermal cells of glandular heads of all species sampled (Figures 11A–E). The intercellular spaces of the epidermal cells contain granular and/or thready material in *V. davidii*, *V. adenoclada*, and *V. chunganensis* (Figures 11A–D).

## Discussion

The trichome types include ribbon, simple and glandular trichomes in representatives of *Vitis* on different vegetative plant organs. These trichomes may play different roles in plant physiology and ecology with variable morphological, mechanical and phytochemical characteristics (Kortekamp and Zyprian, 1999; Wagner et al., 2004).

### Ribbon trichomes

Ribbon trichomes are flattened and twisted with variable densities on the different vegetative organs among species of *Vitis* (Figures 1A,B; Table 1). The young leaves of most *Vitis* species are covered by ribbon trichomes, which may serve as a mechanical barrier against harsh spring temperature, herbivores and pathogens (Kortekamp and Zyprian, 1999; Werker, 2000) and restrict insect activities on the leaf surfaces (Wagner et al., 2004). They may also play a photo-protective role in decreasing light stress (Liakopoulos et al., 2006). Dense ribbon trichomes (Figures 3A–E) may be a significant defense mechanism against abiotic and biotic stresses for young plant organs of *Vitis*. These trichomes may also help decrease water loss and facilitate acclimation to xeric environments (Wagner et al., 2004). During development of these young leaves, in some *Vitis* species sampled (e.g., *V. flexuosa*, *V. rupestris*, *V. rotundifolia*, and *V. shenxiensis*), the ribbon trichomes are deciduous, becoming glabrescent at maturity (Table 1). This phenomenon may be due to the promotion of photosynthetic competence and establishment of phytochemical defense mechanism of leaves when they mature, at which point they no longer need the protection of ribbon trichomes.

Ribbon trichomes are frequently observed in both subgenera of *Vitis* (subgenus *Muscadinia* and subgenus *Vitis*) distributed in North America, East Asia, and Europe (Moore, 1991; Chen et al., 2007; our observations). Interestingly, ribbon trichomes also occur in other genera of the *Ampelocissus*–*Vitis* clade (e.g., *Ampelocissus* and *Pterisanthes*) (Liu et al., 2013, 2016; Wen et al., 2013) in Vitaceae according to the previous studies (Latiff, 1982; Jackes, 1984; Ickert-Bond et al., 2015) and our observations, while, as far as we know they are not observed in any other genera of Vitaceae (e.g., *Ampelopsis, Rhoicissus, Tetrastigma, Cayratia, Cissus, Parthenocissus*, and *Yua*). Our results support that *Vitis, Ampelocissus* and *Pterisanthes* have similar morphological characteristics, confirming the hypothesis that *Vitis* is most closely related to *Ampelocissus* and *Pterisanthes* based on the molecular evidence (Liu et al., 2013, 2016; Wen et al., 2013) and inflorescence morphology (Ickert-Bond et al., 2015). We infer that the ribbon trichome may be a morphological synapomorphy for the *Ampelocissus*–*Vitis* clade, which should be tested in conjunction with a fully resolved phylogeny of the *Ampelocissus*–*Vitis* clade in the future.

### Simple trichomes

In most *Vitis* species, simple trichomes differ in size, density, cell number and growth positon, and they are all unbranched, uniseriate or unicellular (Figures 1J,K). The development of uniseriate trichomes in the majority of flowering plants is achieved by initial enlargement and a series of periclinal divisions of an epidermal cell (Werker, 2000; Yang and Ye, 2013).

A dense indumentum composed of simple trichomes usually acts as a filter protecting plant tissues against damage of ultraviolet-B radiation and as a deterrent to insect activity, including oviposition and feeding in many species (Levin, 1973; Karabourniotis et al., 1995; Liakoura et al., 1997; Manetas, 2003; Yan et al., 2012). Simple trichomes in wine grape leaves (*V. vinifera* L.) are also considered as a defense against photo-damage during leaf development by modifying and filtering both ultraviolet and visible lights (Karabourniotis et al., 1999; Liakopoulos et al., 2006). Kortekamp and Zyprian (1999) demonstrated that the indumentum of grape leaves with both simple and ribbon trichomes acts as a protective barrier and could restrict the development of fungi and prevent the fungal hyphae from reaching the leaf cuticle.

However, the density of leaf trichomes in grapevine cultivars has no influence on the activity and oviposition of leafhoppers, on the contrary, the density of simple trichomes on the leaf veins is negatively correlated with the parasitism rate by *Anagrus* spp., which is a biological control agent to parasitize the eggs of leafhoppers (Pavan and Picotti, 2009). Short simple trichomes did not affect the oviposition of the leafhoppers, but instead reduced the parasitism rate of *Anagrus* spp., while the long simple trichomes could influence the oviposition of the leafhopper on different herbaceous plants (Pavan and Picotti, 2009). We thus infer that different sizes of insects are influenced by the different height of simple trichomes in *Vitis*, with the short simple trichomes (e.g. in *V. davidii* and *V. vinifera*) impeding activities of smaller insects, and the long simple trichomes (e.g., in *V. retordii* and *V. romanetii*) may protect against bigger insects.

### Glandular trichomes and prickles

Glandular trichomes show great variation in morphology and cell structure (Werker, 2000). They may have uni- or multiserate stalks and uni- or multicellular glandular heads. They can be derived from the epidermis (e.g., capitate glandular trichomes) or from both the epidermis and subjacent layers. Some types of glandular trichomes, which are solely derived from the epidermis are found in many species and are particularly prominent in Asteraceae, Cucurbitaceae, Cannabaceae, Lamiaceae, and Solanaceae (Akers et al., 1978; Werker et al., 1985; Nielsen et al., 1991; Ascensão and Pais, 1998; Kolb and Müller, 2004; Tissier, 2012; Celep et al., 2014).

However, all glandular trichomes and the prickles of *Vitis* which were examined histologically in our study are derived from both the epidermis and subjacent layers, which are similar to the glandular trichomes on the leaves of *Drosera* (Fahn, 1979) and those on *Passiflora foetida* stipules (Durkee et al., 1984), as well as the food bodies, which are globose structures attached to the plant by a short peduncle for promoting mutualism between plants (e.g., in Vitaceae) and ants (Buono et al., 2008; Paiva et al., 2009).

Glandular trichomes can secrete terpenes, phenolics, alkaloids, lipophilic or other substances deterring or poisoning herbivores and pathogens (Levin, 1973; Werker, 2000; Tissier, 2012) or secrete resin to protect the developing tissues from cold temperatures (Lapinjoki et al., 1991). Secreted mucilage from digestive glands of *Drosera* acts as a sticky trap for insects (Outenreath and Dauwaldert, 1986).

The glandular trichomes of *V. romanetii* can secret mucilage and emit volatile substance so that many insects are often glued to glands based on our observation (Figures 8A–C). This trichome type may act as a defense mechanism against insects. The composition of mucilage and volatile substance will be explored in future. However, the glandular trichomes of other species lacked apparent secretions.

Akers et al. (1978) reported that accumulation of the secretions of glandular trichomes of *Nicotiana tabacum* can be observed both within extraplasmic and intercellular spaces. These extracellular spaces for the preservation of the secretions were observed in other glandular trichomes as well (Vermeer and Peterson, 1979; Ascensão et al., 1997; Gravano et al., 1998; Turner et al., 2000). In the secretory cells of glandular trichomes of *Passiflora foetida*, dense masses of granular or fibrillar material accumulate in vacuoles, intercellular spaces and beneath the cuticle (Durkee et al., 1984). Similarly, the ultrastructure of the glandular trichomes and the young prickle of *Vitis* show that there are numerous osmiophilic granular, thread-like and irregular masses in the cytoplasm, vacuoles and/or the intercellular spaces in the epidermal cells of glandular heads. These results suggest that the glandular trichomes and the young prickle might excrete substances and the intercellular spaces may be the places of temporary storage of these secretions in the *Vitis* species examined. There is always one stoma at the apex of glandular trichome (except *V. chunganensis*) based on our observations.

Classification of glandular trichomes is based on morphological and ultrastructural differences (Werker, 2000). In *Vitis* the prickles are only present on stems and petioles of *V. davidii*. Considering the morphology, ultrastructural and development of glandular trichomes of other species in *Vitis*, the prickles of *V. davidii* and the glandular trichomes have similar morphological and ultrastructural characteristics and share similar early developmental stages. Our studies reveal that the prickles of *V. davidii* are homologous to glandular trichomes. The prickles undergo early developmental stages like other glandular trichomes (Figures 9A–D, 10A) and then increase in both height and basal width (Figures 9E–G, 10B), lignification (Figure 10C) and harden (Figures 9H, 10D). Our findings are consistent with the developmental process of prickles on raspberries and roses (Kellogg et al., 2011). Furthermore, trichomes as the precursors to prickles have been mentioned in many other species (Delbrouck, 1875; Leelavathi and Ramayya, 1983). The prickles of *V. davidii* may facilitate climbing and deter large herbivores from feeding on this species. Since birds are important seed dispersers of grapes (Hardie and Obrien, 1988), the prickles may protect fruits from frugivores as well.

Glandular trichomes and the prickles are here observed on Asian *Vitis* species, and they do not usually occur on species from North America and Europe. Moore and Wen (2016) suggest that only *V. labrusca* and *V. rotundifolia* occasionally show few glandular trichomes (Table 1). In addition, glandular trichomes are present on the stems of *Ampelocissus martini* and *A*. *arachnoidea* and on the pedicel of *A*. *xizangensis* (Latiff, 1982, 2001; Chen et al., 2007). Conical prickles are also present on the stems of *A*. *aculeata* and *A*. *acetosa* in Papua New Guinea and Northern Australia (Jackes, 1984; Latiff, 2001). Further study is needed to elucidate the origin of the glandular trichomes and prickles of *Vitis* and *Ampelocissus*.

### Taxonomic utility of trichomes in *Vitis*

The trichome types and density of *Vitis* are influenced by different regional environment and growth stages based on our observation (Table 1). For example, not all *V. chunganensis* which were collected from different localities have glandular trichomes. Some plants of *V. piasezkii* only have ribbon trichomes while the others bear simple trichomes along the current years' branchlets. The ribbon trichomes are dense to nearly glabrous on leaves of *V. wilsoniae* during the growing season. The ribbon trichomes with variable density are present on different leaves of *V. balansana*.

Ribbon and simple trichomes are known from most species of *Vitis*, except for some nearly glabrous species (Table 1). Nineteen and three species sampled have dense ribbon and simple trichomes on young leaves respectively, which are useful taxonomic characters. Meanwhile, young leaves of many species often differ in their types and density of trichomes compared to mature leaves on some vegetative organs (Table 1), due to the loss of trichomes during development of leaves. So it is important for identification to examine mature leaves. For example, in *V. wilsoniae*, the young leaves bear very dense ribbon trichomes (Figure 3E), while, there are hardly any ribbon trichomes on the mature leaves (only a few of trichomes on the veins of abaxial leaf surfaces were observed).

Eleven species sampled in this study show a non-deciduous dense arachnoid indumentum composed of ribbon trichomes on the abaxial leaf surfaces, which distinguish these species from any of the other *Vitis* species examined. *Vitis retordii* shows a dense indumentum composed of long simple trichomes on adaxial leaf surfaces, which can easily distinguish this species from any other *Vitis* species examined. Simple trichomes on abaxial leaf vein axils or leaf margins are important taxonomic characters for some species (e.g., *V. flexuosa*, *V. piasezkii*, *V. piasezkii* var. *pagnucii*, and *V. shenxiensis*).

Glandular trichomes and prickles are only documented in seven species and these species can be further distinguished by the morphological differences of the glandular trichomes. The prickles on stems and petioles of *V. davidii* are considered an unique character that distinguishes this species from any other *Vitis* species (Chen et al., 2007). The glandular trichomes of *V. romanetii* are dense, long, pink/yellow and columnar on branchlets, petioles and veins of abaxial leaf surfaces, while those of *V. adenoclada* are dense, long, wine-red and hooked on stems and petioles. In comparison the glandular trichomes of *V. chunganensis* are less dense, short, green and have inverted trapezoid heads on basal veins of abaxial leaf surfaces, petioles and along stem nodes. The distribution of the glandular trichomes of *V. shenxiensis* is very sparse, and the glandular trichomes are short and the glandular heads are oval in *V. shenxiensis*. Details of the distribution of the three types of trichomes in different species in this study (Table 1) can contribute to increase our understanding of *Vitis* systematics and taxonomy.

## Author contributions

ZM and XL designed the study, ZM performed the research and analyzed the data. ZM, JW, SI, LC, and XL discussed the data and wrote the manuscript.

## Funding

This work was supported by the National Natural Science Foundation of China (Grants No. 31370249, No. 31570216).

### Conflict of Interest Statement

The authors declare that the research was conducted in the absence of any commercial or financial relationships that could be construed as a potential conflict of interest.

## Supplementary material

Supplementary Image 1 **The results of the histochemical lignin tests for prickles of different growth stages of *V. davidii*. (A)** Lignin-stained section of a young prickle without lignification. **(B)** Lignin-stained section of a mature prickle with partial lignification on the upper region. **(C)** Lignin-stained section of an old prickle exhibiting complete lignification. Scale bars: 2.5 mm.
