# Supplementary figures and images for: Morphology, Structure, and Ontogeny of Trichomes of the Grape Genus (Vitis, Vitaceae)
Source: Front Plant Sci. 2016 May 25;7:704. doi: 10.3389/fpls.2016.00704 (PMC4879774; doi:10.3389/fpls.2016.00704)

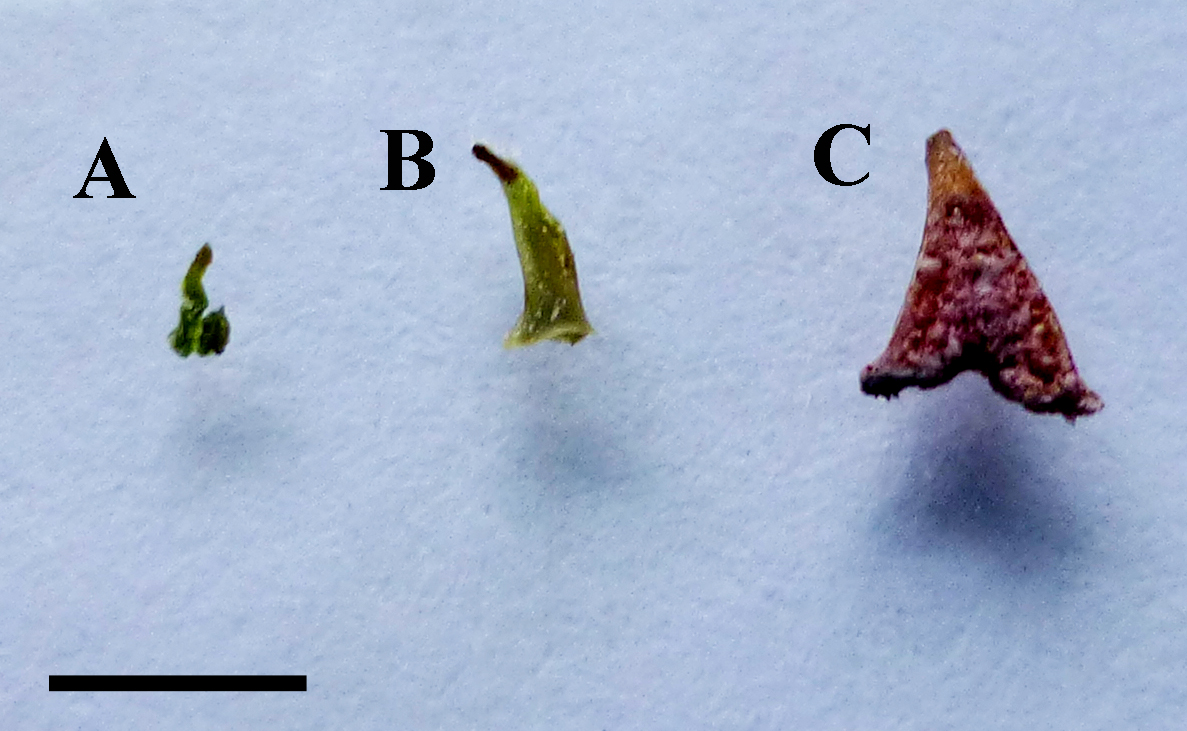

Supplement: Supplementary Image 1 — The results of the histochemical lignin tests for prickles of different growth stages of V. davidii. (A) Lignin-stained section of a young prickle without lignification. (B) Lignin-stained section of a mature prickle with partial lignification on the upper region. (C) Lignin-stained section of an old prickle exhibiting complete lignification. Scale bars: 2.5 mm. [file Image1.JPEG]
